# Comparison between Different Diagnostic Strategies in Low-Risk Reproductive Age and Pre-Menopausal Women Presenting Abnormal Uterine Bleeding

**DOI:** 10.3390/diagnostics10110884

**Published:** 2020-10-30

**Authors:** Chiara Belcaro, Federica Scrimin, Alessandro Mangogna, Emanuele Filippo Galati, Stefania Biffi, Lorenzo Monasta, Federico Romano, Giuseppe Ricci

**Affiliations:** 1 Institute for Maternal and Child Health, IRCCS Burlo Garofolo, Via Dell’Istria 65/1, 34134 Trieste, Italy; chiara.belcaro@burlo.trieste.it (C.B.); alessandro.mangogna@burlo.trieste.it (A.M.); stefania.biffi@burlo.trieste.it (S.B.); lorenzo.monasta@burlo.trieste.it (L.M.); federico.romano@burlo.trieste.it (F.R.); giuseppe.ricci@burlo.trieste.it (G.R.); 2 Department of Obsterics and Gynaecology, University of Verona, 37134 Verona, Italy; emanuelefilippo.galati@studenti.univr.it; 3 Department of Medical, Surgical and Health Science, University of Trieste, 34129 Trieste, Italy

**Keywords:** abnormal uterine bleeding, diagnostic criteria, hysteroscopy, reproductive and pre-menopausal age, endometrial cancer

## Abstract

Abnormal uterine bleeding (AUB) is a common symptom in the female population, with an estimated prevalence of 10 to 30% in fertile age and up to 90% in perimenopausal women. In most cases, AUB is due to a benign cause. However, it can also be a symptom of atypical endometrial hyperplasia or endometrial cancer, a more common disease during menopause which can also affect women in their reproductive age. Considering the high prevalence of this symptom an appropriate diagnostic algorithm is needed. Concerns about the risks, pain, and stress associated with an endometrial biopsy and its impact on the healthcare system make the choice of AUB diagnostic strategy extremely relevant. Even if the scientific community agrees on the definition of AUB, International Guidelines show some differences in the management of women of reproductive age with AUB, especially regarding the age cut-off as an independent indication for endometrial biopsy. This study compared different diagnostic strategies to identify a diagnostic pathway with high sensitivity and specificity but low impact on the health system’s resources. The analysis was based on three diagnostic algorithms defined as part of the guidelines of leading scientific societies. Women of reproductive age with AUB (*n* = 625) and without risk of endometrial cancer were included in the study. Results showed that the best criterion to investigate AUB in women at low risk of endometrial cancer is not age cut-off but the presence or absence of focal endometrial pathology at the ultrasound and the response to the progestin therapy. This approach makes it possible to perform fewer outpatient hysteroscopic biopsies without excluding positive cases from the examination.

## 1. Introduction

Abnormal uterine bleeding (AUB), also known as dysfunctional uterine bleeding, is a common symptom in the female population, with an estimated prevalence of 10 to 30% in fertile age and up to 90% in perimenopausal women [1,2,3]. According to the 2011 criteria by the International Federation of Gynecology and Obstetrics (FIGO), AUB is defined as abnormal bleeding from the uterine cavity by regularity, volume, and timing present for most of the past six months [4]. The FIGO systems have also given an acronym for common etiologies, which apply to chronic AUB. PALM-COEIN is the acronym provided by the FIGO to classify the underlying etiologies of chronic AUB [5]. The first portion, PALM, describes structural issues (P, Polyp; A, Adenomyosis; L, Leiomyoma; M, Malignancy, and hyperplasia). The second portion, COEI, reports non-structural issues (C, Coagulopathy; O, Ovulatory dysfunction; E, Endometrial disorders; I, Iatrogenic). The N stands for “not otherwise classified” [5].

The prevalence of AUB increases mostly in adolescents [6] and perimenopause women [7], when the anovulatory cycles are frequent [8,9]. About 20% of the affected people belong to the adolescent age group, and 50% of the affected individuals are between 40 and 50. It is estimated that about 90% of women present at least one episode of AUB in the pre-menopausal transition period, and 78% of them have a recurrence of at least three episodes [2]. In most cases, AUB is due to a benign cause. However, it can also be a symptom of atypical endometrial hyperplasia or endometrial cancer, a more common disease during menopause which can also affect women in their reproductive age, with an incidence that the National Cancer Institute estimated in 16.7% in the 45–54 age group, and 34.5% in the 55–64 age group [10]. Nevertheless, 5% of women of reproductive age suffer from a malignant pathology [11].

The selection of patients for the endometrial biopsy has the foremost aim of detect endometrial cancer and atypical hyperplasia, for which the early diagnosis is of utmost importance for proper treatment of the disease [12]. It is important to consider that atypical hyperplasia is associated with a concomitant carcinoma in up to 50% of cases [13] and has a risk of neoplastic progression of 12.5% in the following ten years [14]. On the contrary, the histological diagnosis of hyperplasia without atypia is associated with a low risk of neoplastic progression (less than 5% in 20 years) [11,13], and it generally regresses spontaneously during follow-up or responds well to local or systemic progestogen therapy [11,15].

Even if the scientific community agrees on the definition of AUB, International Guidelines show some differences in the management of women of reproductive age with AUB. All guidelines consider the presence of risk factors, such as unopposed estrogen use, tamoxifen treatment, obesity, polycystic ovary syndrome (PCOS), and genetic factors, e.g., families with hereditary non-polypoid cancer of the colon for endometrial cancer, as an independent indication for endometrial biopsy [12,16,17]. But some guidelines consider patient’s age as an independent indication for endometrial biopsy [12,16,17].

Hysteroscopy associated with targeted biopsy is considered the ‘gold standard’ approach for the most accurate evaluation of the endometrium: endometrial hysteroscopic achieves higher sensitivity (78.4–98.0%) and specificity (92.0–95.8%) if compared to blind biopsy [14]. In a previous study, we evaluated the appropriateness of the indications for biopsy to detect endometrial cancer. In women of reproductive-aged, we found that 57% of hysteroscopic biopsies were inappropriate [18]. A consequence of this over-investigation is that many individuals are subjected to the potential harms and costs of treatment without almost none benefits [19].

Since the most frequent indication for endometrial evaluation in reproductive and perimenopausal age is the AUB, it is advisable to establish the optimal management of this symptom in order to decrease the number of inappropriate examinations and to therefore reduce the incidence of complications related to the procedure, such as infection, perforations, visceral injuries vasovagal attack, adverse reaction to anesthetics, and excessive pain [20,21,22,23]. Other than that, inappropriate biopsies are associated with a higher rate of dysfunctional endometrium rather than malignancy. Reducing these procedures allows for an increase in the sensitivity of hysteroscopy for pre-neoplastic and neoplastic lesions [24,25].

The present study aims to compare different diagnostic strategies recommended by the leading scientific societies to manage AUB in patients of reproductive age without risk of endometrial cancer [26,27].

## 2. Materials and Methods

### 2.1. Study Design and Patient Selection

We designed a retrospective cohort study including patients who underwent office hysteroscopy for AUB from January 2012 to December 2014 in the third level hysteroscopy service of the Gynecology Department of Institute for Maternal and Child Health—IRCCS Burlo Garofolo. This study represents a single-center experience.

Women of reproductive age with AUB, defined according to FIGO criteria 2011, were included in the study [4]. Exclusions criteria were: insufficient clinical data, menopause (defined as at least 12 months of amenorrhea), referral due to infertility, cervical disease, isthmocele, pyometra, ongoing endometrial infections, an incomplete procedure for excessive pain, or vagal reaction or risk factors of endometrial cancer: PCOS, ovarian tumors, obesity (body mass index > 30), tamoxifen therapy, family history of endometrial, ovarian, or intestinal cancer, unopposed estrogen therapy, and diabetes. Considering the possible association between endometrial cancer and hypertension described by some authors, we decided to exclude also patients with hypertension [28]. Considering that the sample also includes young women we did not take into account parity, and we considered only infertility as a risk factor. All women underwent hysteroscopically guided biopsy for the histological diagnosis. The study was approved by the Institutional review board (Scientific committee), reference number RC 17-2008, and all participants gave the consent for use their clinical details in scientific researches and scientific publications.

### 2.2. Diagnostic Algorithms Design

**Figure 1:**
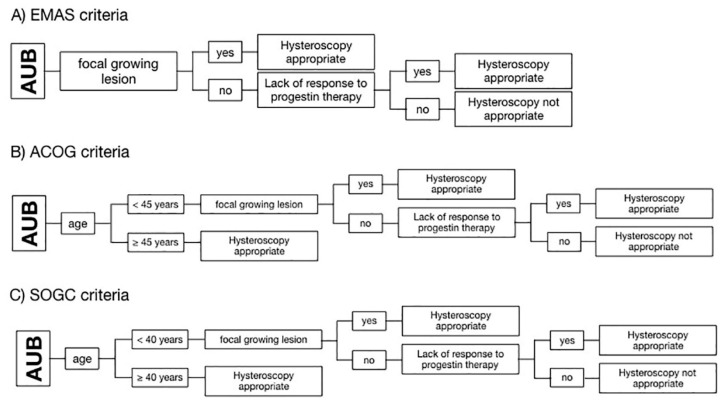
Schematization of the algorithms used to define hysteroscopic biopsy appropriateness according to the criteria of three different guidelines of scientific societies. (**A**) European Menopause and Andropause Society (EMAS); (**B**) American Congress of Obstetricians and Gynecologists (ACOG); and (**C**) Society of Obstetricians and Gynecologists of Canada (SOGC). Diagrams show that ACOG and SOGC consider the age of 45 and 40 years, respectively, as the first criterion of appropriateness.

All patients were evaluated based on three algorithms set up according to the criteria of three different guidelines of scientific societies, which are: (A) the “EMAS clinical guide: assessment of the endometrium in peri e postmenopausal women” of the European Menopause and Andropause Society (EMAS); (B) the “Diagnosis of AUB in reproductive-aged women” of the American Congress of Obstetricians and Gynecologists (ACOG); and (C) the “AUB in premenopausal women” of the Society of Obstetricians and Gynecologists of Canada (SOGC) [12,16,17]. The EMAS algorithm considers hysteroscopy as appropriate if there is a focal growing lesion or in the case of lack of response to the progestin therapy. ACOG and SOGC guidelines add the patient’s age as an independent risk factor: ACOG considers hysteroscopy appropriate for every patient with AUB and ≥ 45 years old, whereas SOGC puts the cut off at ≥ 40 years of age [12,16,17]. Differences between the three algorithms used to define the need for endometrial biopsy are highlighted in Figure 1.

According to the PALM-COEIN classification, we considered as focal growing lesion ultrasound imagines suggestive for polyps or myomas [4]. Regarding the endometrial thickness there is no consensus in the current literature about a cut-off in premenopausal population associated with endometrial cancer, so we did not take into account this value in our analysis [1,29]. The diagnosis were all histological according to WHO 2014 classification. This separates endometrial hyperplasia into two groups based upon the presence of cytological atypia: hyperplasia without atypia and atypical hyperplasia [30]. All the positive exams have been reviewed by two pathologist experts in this field, and there was no discrepancy in the reports.

### 2.3. Statistical Analysis

Descriptive analyses were carried out following the characteristics of the studied variables. We calculated frequencies and percentages, means, and standard deviations or median and interquartile ranges, as appropriate. We calculated sensitivities, specificities, positive predictive values (PPV), negative predictive value (NPV), positive likehood ratio (LR+), and negative likehood ratio (LR-) in detecting endometrial carcinoma, atypical hyperplasia, and atypical polyp. All analyses were conducted with Stata/IC 14.2 (StataCorp LLC, College Station, TX, USA).

## 3. Results

### 3.1. Characteristics of the Study Population

From January 2012 to December 2014, 3036, patients underwent office hysteroscopy for AUB in our center. Following inclusion and exclusion criteria, a study population of 625 women was considered for the analysis. The population characteristics are reported in Table 1.

In 365 (58.4%) patients, a focal growing lesion was detected by ultrasound, whereas, in 260 (41.6%) patients, the imaging evaluation was negative. Histological diagnosis are reported in Table 2.

Of all biopsies, 7 women had a positive histological report of a malignant or premalignant lesion (1.1%) (Table 3).

### 3.2. Application of the Diagnostic Algorithms on the Study Population

**Figure 2:**
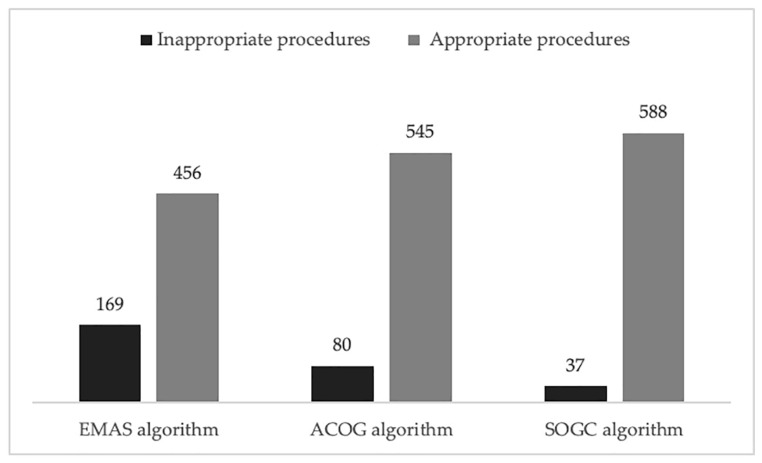
Comparison between different algorithms. The bar chart shows the different number of appropriate and inappropriate hysteroscopies according the three different algorithms considered.

Using the EMAS algorithm, 456 procedures resulted appropriate (73%) and 169 inappropriate (27%). According to the ACOG guidelines, 545 procedures have been classified as appropriate (87%) and only 80 as inappropriate (13%). With the SOGC algorithm, 588 hysteroscopies resulted appropriate (94%) and 37 inappropriate (6%) (Figure 2). Remarkably, no pathological cases were ignored by any of the algorithms, meaning that the sensitivity was 100% for all. What appeared to be different was specificity. Algorithm A had a specificity of 27.3% (95% CI 23.9–31.0%), a PPV of 1.5%, an NPV of 100%, a LR+ of 1.38, and LR- of 0. Algorithm B had a specificity of 12.9% (95% CI 10.4–15.9%), a PPV of 1.28%, an NPV of 100%, a LR+ of 1.15, and a LR of 0. Specificity of algorithm C was 6.0% (95% CI 4.2–8.2%), PPV 1.19%, and NPV 100%, LR+ 1.06, and a LR of 0. By looking at the different guidelines, we could observe that the specificity of algorithm A, assessing the non-response to progesterone, without taking the age as risk factor, was more than double than algorithm B based on the 45 years cut-off and more than four times the algorithm C based on the 40 years cut-off. The confidence intervals of the three results do not overlap. We performed a post-test probability analysis, and we found that the incidence of positive cases was 1.5% with EMAS criteria, while it was 1.3% with ACOG guideline and 1.2% with SOGC criteria.

## 4. Discussion

There is no agreement among scientific societies on the criteria and indications that recommend endometrial evaluation in cases with AUB. The choice of the most suitable path must balance the prevalence of AUB in the population, the risk of cancer in symptomatic patients, and the feasibility and risk associated with diagnostic methods [20,21].

About 90% of women present at least one episode of AUB in the pre-menopausal transition period, and 78% of them have a recurrence of at least three episodes [2]. Our results suggest that up to 50% of patients have dysfunctional endometrium or hyperplasia without atypia probably due to transient anovulatory cycles, as already described in literature [13]. The risk of endometrial hyperplasia without atypia progressing to endometrial cancer is less than 5% over 20 years, and, in premenopausal women, the majority will regress spontaneously during follow-up [13]. Treatment with progesterone has a high disease regression rate.

Endometrial cancer is a typical postmenopausal disease, only 4.5–14% affects premenopausal women. In these women, 5-year survival at stage I is 92–100% [31,32].

In our clinical practice, we choose to perform hysteroscopy associated with targeted biopsy, considered the procedure high sensitivity and specificity [16,24,25]. It is possible to obtain an endometrial sample also with curettage, but this technique loses 11% of malignancy and 60% of atypical hyperplasia, and it carries all the collateral effects related to general anesthesia and complications due to the procedure itself. Endometrial sampling in an outpatient setting presents fewer risks, the complications’ rate is estimated between 1.2% and 3.8% (vasovagal reactions, perforation, and infections) but, even if it is well accepted in the majority of the patients, it is not free from pain [33]. This procedure presents some limitations: in 17% of cases, it is not possible to complete the procedure, and, in 7% of patients, the sample is inadequate for examination [16]. In these cases, could be necessary to perform an operative hysteroscopy in theatre, in order to reduce patient’s discomfort and allow an accurate diagnosis and treatment when possible. The prevalence of complications in operative hysteroscopy is estimated between 0.1 and 1.6% for uterine perforations, 0.02% for visceral injuries (urinary), and 1.9% for endometritis [20,21,33,34]. With a suspect of malignancy, the gold standard is to perform an endometrial sampling after hysteroscopy. A positive hysteroscopy result increases the probability of malignancy from <5% to 71.8%, whereas a negative result reduces the probability to 0.6%. Hysteroscopy in fact is more accurate in detecting endometrial disease rather than excluding it, and the accuracy is higher for endometrial cancer than for endometrial hyperplasia [11,35]. However, we found in a previous study that 57% of requests for hysteroscopy in reproductive-aged women were inappropriate according to the indications of the scientific societies [18].

Considering the high prevalence of AUB in premenopausal women, we have to find the diagnostic strategy that exposes our patients to less unnecessary procedures without losing positive cancer cases [36,37,38].

Based on the prevalence and the natural history of endometrial cancer in fertile age, it may be questionable whether the risks of diagnostic hysteroscopy do not outweigh the benefits [18].

The guidelines of the scientific societies (ACOG, SOGC, and EMA) agree that endometrial biopsy should be proposed to women with AUB who have risk factors for endometrial cancer [12,16,17]. There is also agreement that women who present ultrasound focal growing lesion suggestive of endometrial polyp or submucosal myoma must have direct access to hysteroscopy to avail themselves of the diagnostic and therapeutic treatment and that endometrial thickness in reproductive and premenopausal age, being very variable, cannot be used as a diagnostic criterion [1,12,16,17,29].

Instead, they differ from the criteria for low-risk women without focal lesion. The main difference is the age criterion, considered as an independent risk factor for malignancy and, therefore, an indication for immediate histological evaluation according to ACOG and SOGC algorithm [12,17]. The European guidelines, considering the therapeutic action of progesterone on hyperplasia without atypia, suggest treating all low-risk patients with progesterone and reserving the biopsy only for those who do not respond to treatment, continuing to experience bleeding [16]. In our cohort of patients, adding the age parameter in the diagnostic flow chart seems to increase the numbers of unnecessary procedures, considering the low cancer prevalence in this population [29]. This result is coherent with the recent literature [29,39]. Pennant et al. demonstrates in their review that it is not possible to identify a specific age cut-off in pre-menopausal population above which the risk of malignancy becomes meaningful [40].

Overall, the present study’s findings help to underline and clarify the importance of a progestin therapy trial and the crucial role of ultrasound uterus imaging. In particular, we strengthen the notion that, according to all the examined guidelines, without a recognized intrauterine lesion at transvaginal ultrasound, a trial of progestin therapy is useful to exclude hormonal dysfunctions, avoiding unnecessary invasive intervention. On the other hand, as we have shown in an Italian multicenter study, the presence of intrauterine polyps does not increase the risk of cancer if these are less than 22 mm, but progestin treatment does not resolve AUB in the presence of polyps [41].

Depending on the algorithm used for comparison, between 14% and 21% of biopsies could have been avoided when choosing as an exclusion criterion the response to medical therapy, with no apparent missed diagnosis.

By looking at the different guidelines, we could observe that the specificity of algorithm A, assessing the non-response to progesterone, without taking the age as risk factor, was more than double than algorithm B based on the 45 years cut-off (27.3% vs. 12.9%), and more than four times the algorithm C based on the 40 years cut-off (27.3% vs. 6%). This consequently determines a different incidence of malignancy: 1.5% with EMAS criteria, 1.3% with ACOG guidelines, and 1.2% with SOGC criteria.

Other than that, these findings agree with a previous observation that associated inappropriate hysteroscopic biopsies with a high rate of dysfunctional endometrium (up to 15% of cases), pointing out the importance of being able to rule out any underlying dysfunctional disturbance before the biopsy [11]. Delaying the biopsy of three months to allow a trial of progesterone therapy should not modify the prognosis of patients with endometrial cancer when a focal growing lesion is not detectable [42,43]. Indeed, AUB represents an early symptom of endometrial cancer characterized by a generally slow course that allows making the diagnosis at the initial stages in 70–90% of cases [44,45]. Furthermore, the prognosis of endometrial cancer in young women would appear to be even better than the already good prognosis of women in menopause [31,45,46].

**Figure 3:**
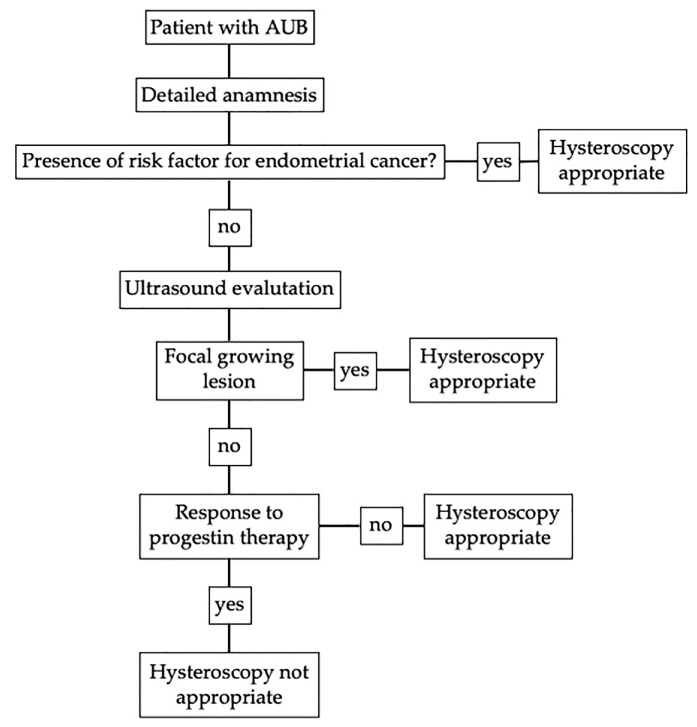
Diagnostic algorithm without age criteria for patient with abnormal uterine bleeding (AUB) in fertile and pre-menopausal age.

To summarize, in case of AUB, an accurate anamnesis is the first necessary step that allows the clinician to exclude risk factors. The recognized risk factors for premenopausal endometrial cancer are PCOS, infertility, ovarian tumors, obesity (body mass index > 30), tamoxifen therapy, family history of endometrial, ovarian or intestinal cancer, unopposed estrogen therapy, diabetes, and hypertension [47]. In women with these characteristics, abnormal uterine bleeding indicates endometrial biopsy according to all the guidelines examined in the study [12,16,17,48]. Laboratory evaluation could be helpful to rule out menopause in some cases. We know the risk of malignancy to be higher in this group therefore a different management is needed. Whereas sex hormones’ level has not been correlated with endometrial cancer, so to know their hematic concentration is not helpful in the management of women with AUB considering also the high variability of sex hormones’ level at this age [49,50]. Thyroid’s disfunction, such as elevated TSH with low fT3 and fT4, and hyperprolactinemia could be associated with AUB, so, if there is clinical suspicious of endocrine disease, laboratory investigation could be useful [51]. After an accurate anamnesis and, if necessary, a clinical-laboratory evaluation, the ultimate decision on whether the biopsy of a patient can be delayed, to investigate the presence of a dysfunctional disturbance, depends on the ultrasound imaging analysis, which, therefore, plays a fundamental role, allowing to exclude the presence of focal growing lesions (i.e., polyps or myomas) that require surgical treatment, as well as ovarian anomalies. As we mentioned before, there is no consensus regarding the endometrial cut-off in premenopausal women associated with endometrial cancer because increase thickness is associated with dysfunctional endometrium, as well [1,29]. In case of negative ultrasound, we suggest three month of progestin therapy, according with all the three guidelines considered. If the patient still has AUB, endometrial biopsy is appropriate. With the suggested algorithm, we still have a high number of false positive cases, but this is acceptable if it does not miss any positive case. The diagnostic algorithm is synthetized in Figure 3.

Therefore, considering the study limitations related to the small sample size, we should concede that we selected a population at low risk of malignancy to compare the three algorithms, with a lower incidence of positive biopsies (1.1%) compared to postmenopausal women, but this data is consistent with the prevalence reported in literature [29,40].

In conclusion, the study shows that in our population, the best strategy to investigate AUB in women at low risk of endometrial cancer does not take into account an age cut-off, but the presence or absence of focal endometrial pathology at the ultrasound and the response to progestin therapy. This approach makes it possible to perform fewer hysteroscopic biopsies without excluding positive cases from the examination. As we suggested in a previous study [18], scientific societies should point out the risks of over-investigation and related overtreatment in their guidelines.

## Figures and Tables

**Table 1:** Population’s demographic characteristics.

	No.	%
**Age**		
<40 years	111	17.7
40–44 years	153	24.5
45–49 years	215	34.4
≥50 years	146	23.4
**Ethnia**		
White-Caucasian	581	93
African	25	4
Asian	13	2
Hispanic/latina	6	1

**Table 2:** Distribution of histological reports.

Histological Diagnosis	No.	%
Atrophic endometrium	18	2.88
Proliferatory/secretive endometrium	68	10.88
Dysfunctional endometrium	94	15
Hyperplasia without atypia	162	26
Myoma	100	16
Polyp	175	28
Other, including fibrotic tissue	1	0.16
Atypical hyperplasia	4	0.64
Adenocarcinoma	3	0.48
Total	625	100

**Table 3:** Clinical characteristics of positive histological reports of malignant or premalignant lesions in the study population.

Patients	Age	Focal Growing Lesion	Failure of Progesterone Therapy	Histological Report
Case 1	55	yes	/	endometrial carcinoma
Case 2	46	no	yes	endometrial carcinoma
Case 3	49	no	yes	endometrial carcinoma
Case 4	41	yes	/	atypical hyperplasia
Case 5	50	no	yes	atypical hyperplasia
Case 6	44	yes	/	atypical hyperplasia
Case 7	44	yes	/	atypical polyp
